# Long-term results of valgus intertrochanteric osteotomy for partial avascular necrosis of the femoral head after femoral neck fracture in adolescents

**DOI:** 10.1186/s12891-023-06598-1

**Published:** 2023-06-05

**Authors:** Antonín Chochola, Jan Bartoníček, Pavel Douša, Michal Tuček

**Affiliations:** 1 Department of Orthopaedics, First Faculty of Medicine, Charles University and Military University Hospital Prague, U Vojenské Nemocnice 1200, 169 02 Prague 6, Czech Republic; 2 Department of Orthopaedics and Traumatology, Third Faculty of Medicine, Charles University and University Hospital Královské Vinohrady, Šrobárova 1150/50, 100 34 Prague 10, Czech Republic

**Keywords:** Femoral neck fractures, Avascular necrosis, Intertrochanteric osteotomy, Long-term results

## Abstract

**Purpose:**

The study evaluates long-term results in patients treated by valgus intertrochanteric osteotomy (VITO) for partial avascular necrosis of the femoral head (ANFH) after fracture of the femoral neck in adolescent age. Although this method is mentioned in literature frequently, there are only few studies in the literature dealing with it in detail.

**Methods:**

Authors evaluated five patients at the interval of 15 to 20 years following VITO. The mean age of the patients at the time of injury was 13.6 years and at the time of VITO 16.7 years. The studied parameters included resorption of necrotic segment of femoral head, development of posttraumatic osteoarthritis and leg shortening.

**Results:**

Comparison of radiographs and MRI scans before and after VITO showed resorption of the necrotic segment of the femoral head and its remodeling in all five patients. However, two patients gradually developed slight osteoarthritic changes. In one patient, remodeling of the femoral head occurred during the first 6 years postoperatively. Subsequently, the patient developed severe osteoarthritis with marked clinical symptoms.

**Conclusion:**

VITO can improve the long-term function of the hip joint in adolescents with ANFH after a femoral neck fracture, but cannot restore completely the original shape and structure of the femoral head.

## Introduction

Femoral neck fractures in children and adolescents are rare injuries, often associated with a number of complications. The most severe of these is avascular necrosis of the femoral head (ANFH) [1–9]. The published studies describe multiple methods of its treatment, ranging from core decompression up to total hip replacement [7, 10–14]. One of these methods is intertrochanteric osteotomy (ITO) based on the principle of reorientation of the necrotic segment of the femoral head away from the weight-bearing zone of the hip joint [15–17]. Although this method is mentioned quite frequently, there are only a few studies in the literature dealing with it in detail, mostly in the form of case reports, with the follow-up period not exceeding seven years [14, 15, 18–20]. Moreover, a number of important facts have been neglected there. The aim of this article is to present some long-term clinical and radiological results of valgus intertrochanteric osteotomy, as well as a literature review on this issue.

## Material and methods

### Study group

Between 1999 and 2006, we performed valgus intertrochanteric osteotomy (VITO) in six patients for partial ANFH after fracture of the femoral neck. Inclusion criteria were partial ANFH after fracture of the femoral neck in adolescent age verified by MRI, varus deformity of proximal femur and the leg shortening minimally of 2 cm. The results were published in 2012 [14]. In 2021, five of these patients with their informed consent (two male and three female), with a minimal follow-up period of 15 years, were examined (Table 1). The mean age of these patients at the time of injury was 13.6 years (range, 12–19) and at the time of VITO 16.7 years (range, 14–21); the mean injury-VITO interval was 36 months (range, 24–76). The average follow-up was 17 years (range; 15–20). The primary injury was caused in one case by a car accident, in two cases by a fall whilst skiing, in one case by a fall from a tree, and in one case by slipping on a wet floor. All patients sustained a transcervical fracture (Colona Type II), that was undisplaced in two cases (N1 and N4). Two patients (N1 and N4) were treated nonoperatively, internal fixation by lag screws was used in three patients (N2, N3 and N5). All patients developed partial ANFH, Ratliff type 2. Except for the oldest female patient (N5), physes of the injured proximal femur were open in all patients at the time of injury. At the time of osteotomy, physes in patients N3, N4 and N5 were closed in both the affected and the contralateral intact hips. In patients N1 and N2, physes in the contralateral proximal femur were open. In the female patient N2, only the greater trochanter physis was open in the affected femur; the femoral head physis was closed. In patient N1, the greater trochanter physis and the medial part of the femoral head physis were open, while the lateral part of the femoral head physis was damaged.

**Table 1:** Basic data on patients

**Pt**	**Fracture type**	**Fracture Therapy**	**Injury** **age** **(y)**	**VITO** **age** **(y)**	**Interval Injury—-VITO** **(m)**	**LLD** **before** **VITO** **(cm)**	**LLD** **Final** **(cm)**	**Shelf arthroplasty**	**FU** **(y)**	**Final result**
**N1** **M**	transcervical undisplaced	Nonoperative treatment	12	14	24	2	1	-	16	very good
**N2** **F**	transcervical displaced	Lag screws	12	14	24	2	1	54 m after VITO	16	good
**N3** **F**	transcervical displaced	Lag screws	12	18	72	2	0	-	20	good
**N4** **M**	transcervical undisplaced	Nonoperative treatment	13	16	36	2	0	19 m after VITO	19	fair
**N5** **F**	transcervical displaced	Lag screws	19	21	24	1	0	-	15	bad

*Pt* patient, *N* number of patient, *M* male, *F* female, *VITO age* age at the time of valgus intertrochanteric osteotomy, *LLD* leg length discrepancy before VITO, *LLD Fin* final leg length discrepancy, *FU* follow up, *y* year, *m* month

### Methods

The indication for intertrochanteric osteotomy was ANFH involving the weight-bearing zone of the femoral head, in four cases associated with shortening of the affected lower limb by 2 cm. Radiographs of the pelvis in AP and Lauenstein projections and AP view of the affected hip were obtained in all the patients. ANFH could be clearly seen on the radiographs in all five cases. The patients also underwent MRI examination where the coronal and sagittal scans served to assess the extent and localization of the necrosis. In all cases, coronal MRI scans showed 50% to 66% involvement of the femoral head and sagittal scans 30% to 50% involvement of the femoral head. These findings served as a basis for preoperative planning, with the aim to transpose the intact part of the femoral head to the weight-bearing zone and to resolve the limb shortening. In all the mentioned cases, our treatment of choice was VITO. All operations were performed by the senior author (JB).

### VITO operative technique

**Fig. 1:**
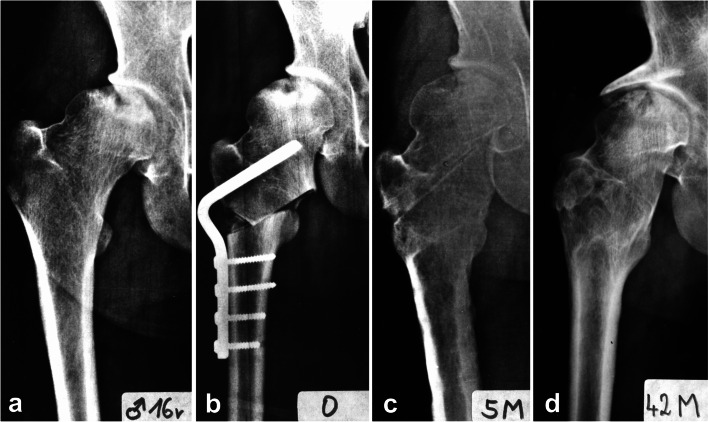
Principles of operative treatment. **a** Patient N4, 16 years old, with polar ANFH; **b** valgus intertrochanteric osteotomy; **c** healing of osteotomy after five months; **d** hip joint 42 months after osteotomy and 23 months after Bosworth hip shelf arthroplasty

This procedure was performed in the same manner as described in detail for adults [14, 16] under control of image intensifier. Preoperative planning, based on radiographic examination, was crucial to the success of the surgery. At surgery, the patient was placed in a supine position, and a lateral longitudinal approach was used. After elevating the proximal portion of the vastus lateralis muscle anteriorly, an L-shaped anterior arthrotomy, running in the direction of the lesser trochanter was done. This improved visualization for the introduction of the implant blade and made it possible to release the insertion of the medial part of the capsule in the area of the lesser trochanter, including the iliopsoas tendon. The osteotomy line was made parallel to the inserted chisel. The wedge angle was determined on the basis of preoperative measurement on MRI scans and ranged between 30 and 40 degrees. After the removal of the seating chisel, the blade of a 120-degree angled blade-plate was inserted into the proximal fragment. In patients with an open physis of greater trochanter the blade-plate had to be inserted in such a way to prevent damage to the physis and, consequently, potential arrest of the growth of the greater trochanter. This required modification of a 120-degree plate to a 140-degree plate. Subsequently, a careful valgus reduction of fragments was done with the limb in abduction. The plate was then fixed to the femoral shaft, limb length and range of motion in the hip joint were examined, drains inserted, and the wound closed (Fig. 1).

### Postoperative management

AP and lateral radiographs of the operated hip were obtained. Postoperative follow-ups, including radiographic examination, were scheduled for 6 weeks, 3 months, 6 months and 1 year. During the initial 6 postoperative weeks, the patient walked with crutches, without weight-bearing of the operated limb. Partial weight-bearing was permitted 6 weeks after surgery and full weight-bearing after three months. Hardware was removed within one year after osteotomy, once union was assured. MRI examination was performed in all patients two to three years after the osteotomy, based on which resorption of the necrotic segment was assessed.

### Additional procedures

In two patients (N2 and N4) with acetabular dysplasia that was present already before the osteotomy, Bosworth shelf arthroplasty was performed, following the original descriptions, 54 months (N2) and 19 months (N4) after VITO [21]. These patients were operated on in the supine position, via the Smith–Petersen approach. Close above the attachment of the superior articular capsule of the hip joint, an arch-shaped notch 3-cm wide and 5-cm long, was made with a chisel, passing obliquely proximo-medially as far as the inner cortex of the iliac bone. A 4 × 5 × 5 × 3 cm monocortical bone graft, harvested from the iliac wing, was firmly hammered into the notch. This self-locking extra-articular graft covered the superior aspect of the femoral head sufficiently without limiting the range of movement in the hip joint (Fig. 1). The postoperative protocol was the same as for intertrochanteric osteotomy.

### Postoperative assessment

All patients were followed up over the whole period by the senior author (JB). The mean follow-up period was 17 years (range, 15–20). Subjective assessment was based on the patients’ satisfaction with the surgery. Objective assessment included VITO healing time, limb lengthening, functional results and potential complications.

## Results

An overview of the results is presented in Table 1. The surgical wounds healed without complications in all patients. The intertrochanteric osteotomies healed in all patients within three months, except for female patient N3 in whom nonunion occurred, requiring treatment five months after osteotomy. No complications were encountered after Bosworth hip-shelf arthroplasty.

### Leg shortening

Leg shortening was fully corrected by VITO in all five patients. In two patients (N1, N2) operated on at the age of 14 years, with an open capital physis of the contralateral femur, the continued growth of the unaffected contralateral femur resulted in a final shortening of the operated leg of 1 cm.

### Resorption of necrotic segment

**Fig. 2:**
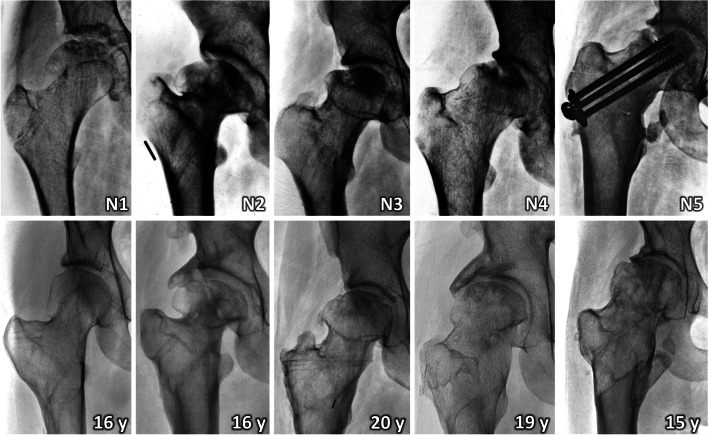
Final results 15 – 20 years after valgus intertrochanteric osteotomy. N – number of patient (see Table 1), y – year

**Fig. 3:**
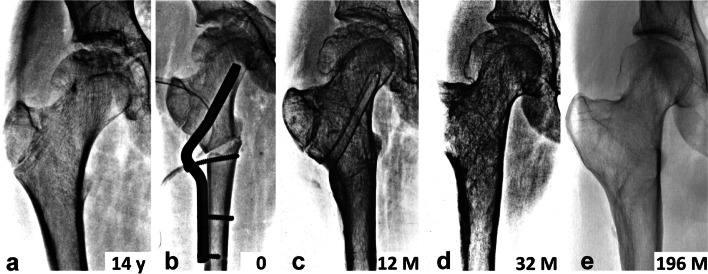
Remodeling of proximal femur in patient N1. **a** hip joint two years after an undisplaced transcervical fracture of proximal femur, treated non-operatively, with polar ANFH; **b** 40-degree valgus intertrochanteric osteotomy with a modified 120-degree angled blade plate, physis of greater trochanter was preserved; **c** the result 12 month after surgery; **d** complete remodeling of proximal femur 32 months after surgery; **e** the final result 16 years after surgery

**Fig. 4:**
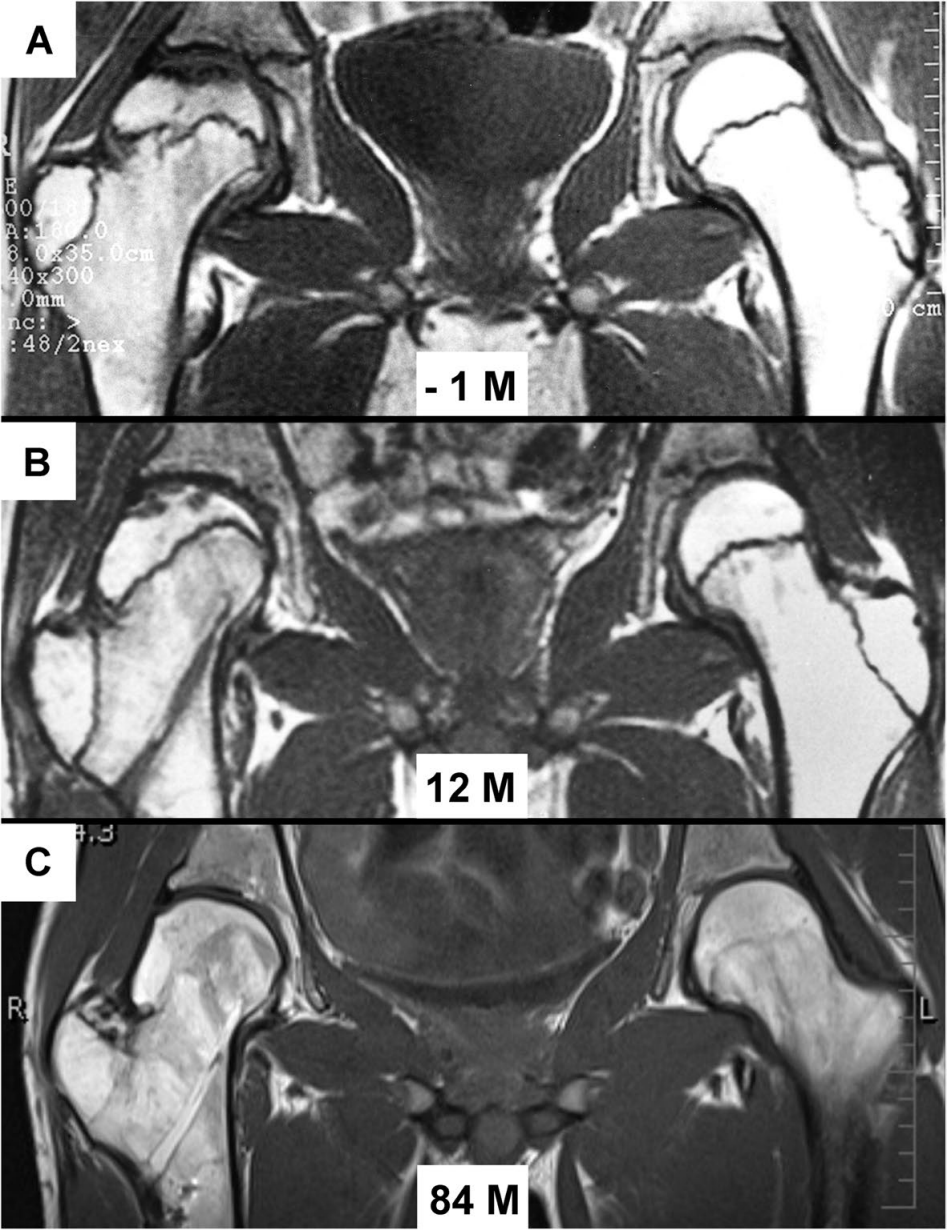
Remodeling of proximal femur in patient N1 on MRI. **a** hip joint one month before surgery; **b** situation 12 months after surgery, with a clearly visible change in the shape of capital physis of injured femur; **c** situation 84 months after surgery

**Fig. 5:**
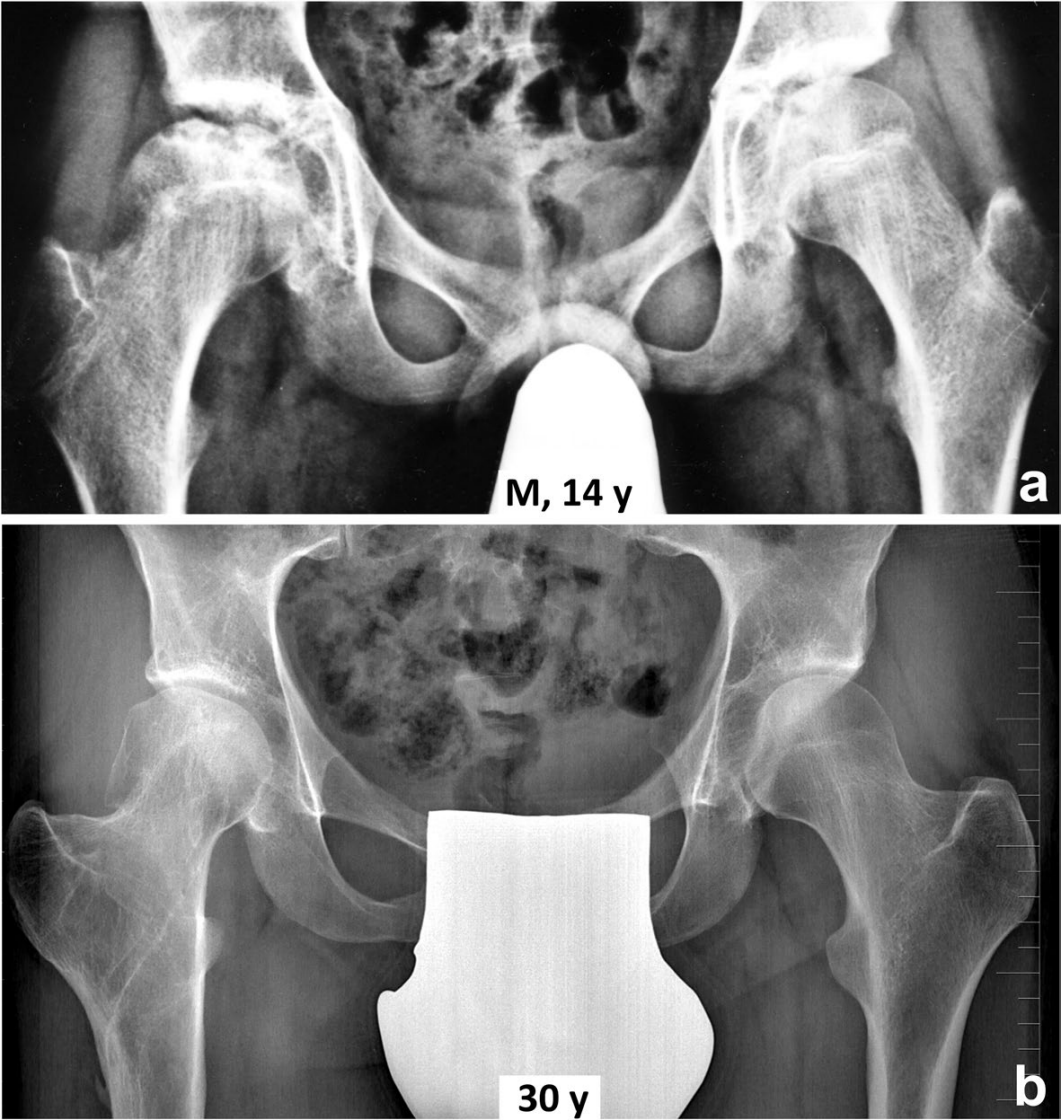
Patient N1 – the final result. **a** pelvic radiograph before surgery, **b** pelvic radiograph 16 years after surgery

Comparison of radiographs and MRI scans before and after VITO showed resorption of the necrotic segment of the femoral head and its remodeling in all five patients (Fig. 2). In patient N1, remodeling of both the femoral head and the entire proximal femur was observed (Figs. 3, 4 and 5).

### Development of posttraumatic osteoarthritis

**Fig. 6:**
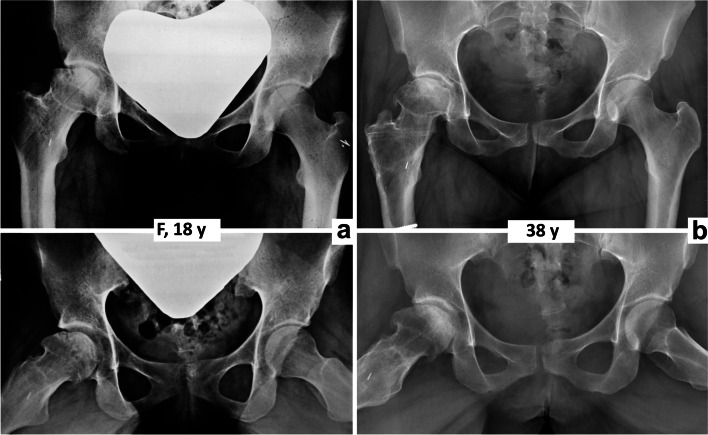
Patient N3 – the final result – in pelvis anteroposterior and Lauenstein views. **a** before surgery; **b** 20 years after surgery

**Fig. 7:**
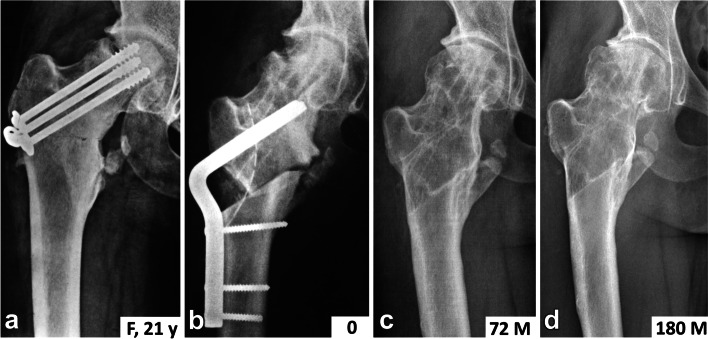
Patient N5 – the final result. **a** hip joint before surgery; **b** after surgery; **c** the result 6 years after surgery; **d** – progression of osteoarthritis 15 years after surgery

Two patients (N3, N4) gradually developed slight osteoarthritic changes, although with minimal symptoms (Figs. 1 and 6). In one female patient (N5), remodeling of the femoral head occurred during the first six years postoperatively. Subsequently, her condition became gradually worse and the patient developed severe osteoarthritis with marked clinical symptoms (Fig. 7).

### Subjective assessment

All patients assessed the effect of operation highly positively. At the final follow-up patient N1 had no complaints, female patients N2 and N3, each a mother of two children, reported slight pain after a higher physical load (walking 10 km). Patient N4 experienced occasional pain after normal physical load. Female patient N5 had permanent pain of varying intensity; she was offered total hip replacement, but rejected it.

### Objective assessment

Patient N1 had a full, pain-free range of motion in the hip joint. In female patient N2, rotation was limited by 10 degrees as compared to the intact limb, and in female patient N3, rotation was limited by 15 degrees, with slight pain in extreme positions. In patient N4, flexion was limited by 10 degrees as compared to the intact limb and rotation was only in the range of 10 degrees. Female patient N5 had a flexion of 0–90 degrees and completely limited rotation. Patients N1 and N2 showed a 1 cm shortening of the limb.

### Complications

Female patient N3 developed a biologically active non-union after VITO, due to early full weight-bearing, which was treated by refixation with a condylar plate and a lag screw and healed two months after the reoperation.

## Discussion

ANFH after a femoral neck fracture in children and adolescents is associated with limb shortening and deformity of the proximal femur, i.e., shortening of the femoral neck, overgrowth of the greater trochanter resulting in post-traumatic varus, reduced articular-trochanteric distance and limb shortening. VITO allows both a reorientation of the necrotic segment and, at the same time, a change of the shape of the proximal femur, with lengthening of the shortened limb. Various techniques of valgus intertrochanteric osteotomy have been described in the literature [17, 22, 23]. We prefer the classical technique with the use of a 120-degree angled blade-plate, which allows a lateral shift of the femoral shaft and, if need be, flexion/extension of proximal fragment [14, 16, 24, 25].

Of great importance for indication of intertrochanteric osteotomy (ITO) in partial ANFH are preoperative MRI scans, which serve for an exact determination of the size of the necrotic segment and its localization. These details are essential for the choice of the osteotomy type (varus/valgus), the wedge size, or the valgus angle. Based on localization of the necrotic segment in sagittal MRI scans, valgus (varus) osteotomy may be combined with flexion/extension osteotomy in the sagittal plane. This was the case of patient N1, where VITO was combined with a 15-degree flexion osteotomy. From this viewpoint, the Ratliff radiological classification of posttraumatic ANFH is inadequate, as it recognizes neither the size nor the location of the necrotic segment of the femoral head in partial ANFH [26]. However, an MRI-based classification is still missing in the literature.

Multiple studies in the literature deal with the results of intertrochanteric osteotomy for ANFH, however, only a few of them focus on ANFH after femoral neck fractures in children and adolescents [14, 15, 18–20].

Boitzy [18] used valgus intertrochanteric osteotomy in 11 patients after a proximal femoral fracture: twice in case of varus malunion, once in varus nonunion, in five cases of ANFH and in three cases of varus nonunion associated with ANFH. Average patient age at the time of injury was 14 years (range, 8–20) and at the time of osteotomy 20 years (range, 9–33). The follow-up was limited to one to three years. In patients with ANFH, the result was fair, or poor. In one case, it was necessary to perform arthrodesis of the hip after the osteotomy.

Forlin et al. [19] reported complications in a group of 16 children after proximal femoral fractures. The average patient age at the time of injury was 12 years (range, 5–16) and average follow-up seven years (range, two to 24). A total of 5 patients developed ANFH Ratliff type 2 [26]. Only 1 patient was treated with varus osteotomy for this diagnosis 2 years post-injury. After 10 years, the patient showed a marked varus deformity of the proximal femur requiring revalgization. The result was assessed as fair.

Nötzli et al. [15] described partial ANFH in three patients aged 13, 14 and 17 years. All patients underwent intertrochanteric extension osteotomy. The follow-up period was three, three and a half, and seven years, respectively. In two cases, the condition improved; in the third case, the femoral head was distorted. Leg shortening after osteotomy was 2.0, 2.5 and 2.5 cm, respectively.

Abbas et al. [20] described three patients 12, 14 and 15 years old, after a transcervical fracture of the femoral neck, who underwent rotational transtrochanteric osteotomy for polar necrosis of the femoral head. The follow-up period was two, two and two and a half years, respectively. Limb length was not specified. Good results were achieved in all three cases.

The problem of all these studies was a small number of patients and in a majority of cases a short follow-up period [14, 15, 18–20]. No author, except for Abbas et al. [20], mentioned the injury-osteotomy interval. In the Abbas´ patients, osteotomy was performed 6 to 11 months post-injury, while in our series, 2 to 7 years post-injury as the patients were initially treated in other medical centers. Based on our experience and data from literature, we believe that the interval between detection of ANFH and osteotomy, together with the patient´s age and status of physes, is an important factor influencing the final outcome. The sooner the operation and the greater the remodeling potential of proximal femur, the better results can be expected. This has been confirmed by very good results achieved in our patients N1 and N2 who were operated at the age of 14 years. By contrast, the female patient N5 operated on at the age of 21 years showed the worst result.

In this context it is interesting to compare the state of physes in the patients´ proximal femurs. Patients N3, N4 and N5 had the physes closed in both proximal femurs before VITO; there occurred only remodeling of the femoral head, without any change in the limb length. Patients N1 and N2 had both physes (femoral head, greater trochanter) open in the intact hip. On the affected side, the female patient N2 had only the physis of the greater trochanter open which, however, fused 3 months after VITO. In patient N1, the greater trochanter physis and the medial part of the femoral head physis were open, while the lateral part of the femoral head physis was damaged. This patient showed after VITO a marked remodeling of the injured proximal femur (Fig. 3) including the capital physis (Fig. 4). In the female patient N2 there occurred only remodeling of the femoral head. In both patients (N1, N2) the intact femur kept growing for another 4 years after VITO and both physis of the proximal femur were closed at the age of 18 years, as documented also by the postoperative follow-up. During this period the intact leg became longer by 1 cm, while immediately after VITO both limbs were of the same length in both patients. According to Ogden [27], physes of the proximal femur close at the age of 16 to19 years, which corresponds with our finding.

Of great importance is, of course, the size and location of the necrotic segment. For these reasons, children and adolescent patients with a femoral neck fracture should be followed up regularly for a long period. In case of a suspected proximal femoral growth disturbance or ANFH, MRI should be performed. Early detection offers the possibility of a timely and optimal treatment. Unfortunately, this is not often the case. For VITO it is necessary to take into account also the growth potential of the contralateral proximal femur. This was the case of our patients N1 and N2 who, despite limb length correction, showed limb shortening as a result of subsequent growth, fortunately merely by 1 cm.

In our present study, the minimal follow-up is 15 years, which has no parallel in the literature In all our 5 patients, VITO considerably improved the function of the hip joint; only in one female patient (N5) did the condition deteriorate over 15 years postoperatively.

A disadvantage of this study is a small number of patients, which does not allow a detailed analysis of the factors influencing the VITO result; nevertheless, the best results seem to be associated with younger patients with a preserved growth potential. In these patients with open physes of the contralateral proximal femur, however, it is necessary to take into account a potential relative shortening after lengthening of the affected limb.

Our results show that a properly indicated, planned and performed VITO can considerably improve the function of the hip joint in adolescents with ANFH after a femoral neck fracture in the long term, but cannot restore completely the original shape and structure of the femoral head. However, additional studies with adequate numbers of patients and long-term follow-up will be necessary in order to confirm these conclusions.

## Data Availability

Basic data on patients are presented in the text, tables and figures. Obtaining more detailed data, for example complete radiological documentation, is possible for reasonable reasons after agreement with the corresponding author.

## Abbreviations

ANFH: Avascular necrosis of the femoral head
ITO: Intertrochanteric osteotomy
MRI: Magnetic resonance imaging
VITO: Valgus intertrochanteric osteotomy
